# Report of Four Cases Suggesting Status Lymphaticus

**Published:** 1913-01

**Authors:** 


Report of Four Cases Suggesting Status Lymphaticus. J. A. Goodrich, M. D., Des Moines. *Ia. Med. Journal,* Oct., 1912. The death of four children in one family within a period of three months and four days and all occuring under similar circumstances presented a condition of more than ordinary interest.

The father of these children is alive and well; the mother died of heart and lung affection, in October, 1910, some eighteen months previous to the death of the first child. In January, 1912, the father again married. In the father's family tree, we find the death of eight of his brothers and sisters, all under nine years of age; three from Scarlet Fever, one Erysipelas, one Flux and three unknown. On the mother's side we find nothing of unusual interest.

Case 1. April 8, 1912, Wyetta, aged four, suddenly died. For a few weeks some impairment of hearing had been noted, and her breathing had been somewhat difficult and stertorous, but this was ascribed to a cold and was treated with home remedies. About a year previously this child was afflicted with diphtheria and was given 5000 units of antitoxin with recovery, but she seemed somewhat more sluggish after this time. During the few days before her death this apathy was more marked. She did not care to play, was afflicted with enuresis, nocturnal and diurnal. At about midnight the night before her death she aroused her father by an attack of choking followed by vomiting, after which she slept till morning. On arising she was noticed to have a pallid look.

She again vomited and was put back to bed, and a physician was summoned, but Dr. Kelley found her dead on his arrival.

Case 2. Eight days following the death of Wyetta, Cecil, a bright little two-year old girl fell down the cellar stairs, fracturing her left clavicle. This I dressed that evening. At this time she seemed bright and sat up in bed while I applied the dressings. The following morning at about seven she arose and seemed quite well, eating only a little cake for breakfast, however. Soon after eight o'clock she was seized with a spasm, which was soon followed by another. Then she said she was hungry and was given some strawberries, of which she ate quite heartily. She was then put to bed and was soon asleep. This did not last long however for she was soon heard screaming. By the time her mother reached her, she was dead.

Case 3. June 7, Walter, aged five and one-half years, died just as suddenly and mysteriously as did the others. Some two weeks prior to this time he, like Wyetta, was noticed to be able to hear less acutely than usual. His tonsils and adenoids, as well as all superficial lymphatic glands, were noted to be hypertrophied. Syrup of ferrous iodide was given in full doses. On this morning he had seemed to be in his usual health and had gone out into the garden and was picking potato bugs off the potatoes when he was called because the sunshine seemed to be rather hot. He went into the house and lay down on the bed but said he was not sick. Soon after this, on going to his room he was found dead. One arm had been raised over his head and a little bloody froth exuded from the mouth. His face, as did the others, bore a mottled look. This child had experienced a little excitement earlier in the day. When his sister had returned from school with her promotion card, this being the last day of school, he became much interested and could talk of little else than of what he would do when he entered school this fall.

Case 4. On July 12, just three months and four days from the death of Wyetta, Josephine, aged seven, the last of the family of four happy and contented children, was stricken. Since the death of Cecil and more especially of Walter, she had been watched very carefully, not only by the family but by physicians as well. She, like Wyetta and Walter, suffered with enlarged tonsils, and during her last few days of life the warning deafness appeared. On two or three occasions she was seized with spells in which she thought she was being choked, but recovered from them. In one of these seizures she said, "Oh, my God, someone is choking me." She then turned cyanotic and sweat freely. After recovery she asked if her mother had seen all the people around her.

During the night previous to her death, she had become greatly alarmed because of the thunder storm then raging, but after its cessation, she slept quite well. At seven her father, on going to work awakened her and asked her how she felt, as was his custom. She replied "all right," and returned to sleep. Some two hours later she arose and went to the living room and started to dress. Her head was then noted to be inclined on her chest, and her face mottled and cyanotic. Attempts to revive her failed, and death followed immediately.

